# Supplementary material for: The Role of Macrophages in Liver Fibrosis: New Therapeutic Opportunities
Source: Int J Mol Sci. 2022 Jun 14;23(12):6649. doi: 10.3390/ijms23126649 (PMC9224467; doi:10.3390/ijms23126649)
Supplement: Supplementary file 1 [file ijms-23-06649-s001.zip › ijms-1743726-Supplementary.pdf]

International Journal of
Molecular Sciences

MDPI

Table S1: Macrophages surface markers and their functions.

Surface markers	Functions of the surface marker	References
CD11	Cluster of differentiation 11 (CD11) protein is a heterodimer complex that consists of CD11 and CD18. It is involved in numerous adhesion-related associations between cells such as monocytes, macrophages, natural killer cells, and granulocytes. It also regulates the uptake of complement-coated particles within cells.<br>- CD11a/CD18 (namely LFA-1) is distributed in all leukocytes.<br>- CD11b/CD18 (namely Mac-1) is expressed by monocytes, macrophages, some lymphocytes, natural killer cells, neutrophils, granulocytes.<br>- CD11c/CD18 (namely p150,95) is a surface marker of monocytes, granulocytes, macrophages, NK cells, and certain lymphocytic tumor cell lines.<br>- CD11d/CD18 is distributed in monocytes, macrophage foam cells, splenic red pulp macrophages, and lymphocytes.	[83,84]
F4/80	The F4/80 glycoprotein, also known as Ly71 and EMR1, has been established as one of the most specific cell-surface markers for mouse mononuclear phagocytes. It is highly and constitutively expressed on most resident tissue macrophages, including the red pulp macrophages, microglia, KCs, and Langerhans' cells. High expression of F4/80 has been proposed as a marker for populations of mouse tissue macrophages that derive from embryonic progenitors and renew independently of blood monocytes [85]. It shares 68% amino acid identity with human EGF-like module-containing mucin-like hormone receptor-like 1 (EMR1) [86-87].<br>Warschkau and Kiderlen demonstrated that the administration of the anti-F4/80 antibody inhibits the production of cytokines (namely TNF-a, IL-12, and IFN) from whole spleen cell cultures of SCID mice exposed to heat killed *Listeria monocytogenes* [89].	[85–89]
Clec4f	C-type lectin domain family 4, member F (Clec4f) is a heavily glycosylated membrane protein co-expressed with F4/80 on KCs. In contrast to F4/80, Clec4f is detectable in fetal livers at embryonic day 11.5 (E11.5) but not in yolk sac, suggesting the expression of Clec4f is induced as cells migrate from yolk cells to the liver [90,91]. Biological functions of Clec4f have not been elucidated. However, researchers have identified that:<br>- KCs express multiple endocytic lectin receptors including a C-type lectin Clec4f. Clec4f recognizes desialylated glycans with an unclear *in vivo* role in mediating platelet destruction [92].<br>- in KCs, ClecC4f interacts with alpha-galactosylceramide (α-GalCer) in a calcium-dependent manner and participates in the presentation of α-GalCer to natural killer T cells [90].	[90–92]
CD68		[93,94]

	Cluster of differentiation 68 (CD68) is predominantly expressed in late endosomes and lysosomes of macrophages, but is also found on the surface of dendritic cells and osteoclasts. CD68 has been widely used as a pan-macrophage marker [93].<br>The function of CD68 is poorly investigated but its preferential location within late endosomes may suggest a role in peptide transport/antigen processing [94].	
CD14	Cluster of differentiation 14 (CD14) one of the first identified Pattern Recognition Receptors (PRRs), is a glycosylphosphatidylinositol (GPI)-anchored receptor known to serve as a co-receptor for several toll-like receptors (TLRs) both at the cell surface and in the endosomal compartment. CD14 plays multiple roles in microbial recognition and signaling of LPS and host-pathogen interactions.	[95]
Ly6C	Ly-6C^{hi} monocytes express the chemokine receptor CCR2 and rapidly infiltrate tissue upon injury, while Ly-6C^{low} monocytes express higher levels of CX3CR1 and show a patrolling behavior *in vivo*.<br>Intravital microscopy experiments in mice suggest that the Ly-6C^{low} monocytes crawl along the hepatic endothelium, possibly to orchestrate the disposal of apoptotic cells or other cellular debris.<br>Upon acute or chronic hepatic injury in mice, Ly-6C^{hi} blood monocytes are massively recruited to the liver and predominate the macrophage pool. These monocyte-derived macrophages initially exert pro-inflammatory and profibrogenic actions, they can differentiate into Ly-6C^{low} monocyte-derived macrophages, which may foster tissue repair and injury resolution.	[60]
CSFR1	Colony stimulating factor 1 receptor (CSF1R), also known as macrophage colony-stimulating factor receptor (M-CSFR), and cluster of differentiation 115 (CD115), controls the proliferation, differentiation, survival and function of macrophages.	[96]
